# The first case of balloon-occluded retrograde transvenous obliteration and partial splenic embolization for gastric varices in situs inversus abdominus

**DOI:** 10.1007/s12328-020-01332-z

**Published:** 2021-01-05

**Authors:** Yusuke Watanabe, Akihiko Osaki, Nobuo Waguri, Shuji Terai

**Affiliations:** 1 Division of Gastroenterology and Hepatology, Niigata City General Hospital, Niigata, Japan; 2 Division of Preemptive Medicine for Digestive Disease and Healthy Active Life, School of Medicine, Niigata University, 1-757 Asahimachi-dori, Chuo-ku, Niigata, 951-8510 Japan; 3 Division of Gastroenterology and Hepatology, Graduate School of Medical and Dental Sciences, Niigata University, Niigata, Japan

**Keywords:** Balloon-occluded retrograde transvenous obliteration, Gastric varices, Partial splenic embolization, Polysplenia, Situs inversus abdominus

## Abstract

Situs inversus abdominus is a congenital abnormality characterized by the mirror image positioning of the abdominal organs, making it difficult to diagnosis and treatment of many diseases. Here, we report a rare case of gastric varices in situs inversus abdominus patient. A 55-year-old man was diagnosed with F2-shaped gastric varices associated with alcoholic cirrhosis. Abdominal dynamic computed tomography showed situs inversus abdominus, and gastric varices with a gastrorenal shunt. Due to the complex anatomy, emergency interventional radiology during bleeding was extremely difficult. Therefore, after thorough consultation, we decided to treat gastric varices prophylactically by balloon-occluded retrograde transvenous obliteration. After treatment, the patient’s clinical course was generally good, with no adverse events, and the gastric varices disappeared. It is important to assess anatomical positioning using three-dimensional reconstruction computed tomography images before treatment, to adequately prepare, including selecting appropriate devices (i.e., catheters and sheath). Although preventive treatment of gastric varices is controversial, in cases with anatomical complexity, preventive treatment with sufficient preparation would be recommended. To the best of our knowledge, there are no reports of treatment for gastric varices in situs inversus abdominus patient. This case will serve as a reference for successful treatment in future cases.

## Introduction

Gastrointestinal varices are often caused by hemodynamic changes that compensate for portal hypertension. Depending on the grade of esophagogastric varices, one in four cirrhotic patients will experience bleeding or rupture events within 2 years of the appearance of varices, leading to a high mortality (20–35%) [1]. Although bleeding from gastric varices (GVs) is less frequent than from esophageal varices (EVs) [2], the mortality rate during bleeding from GVs is high (45–55%) [3, 4]. Therefore, controlling GVs and preventing their rupture is important. The indication for preventive treatment of GVs remains controversial; however, many medical centers have approved GVs for F3-shaped varices, the presence of red color signs, and for F2-shaped varices with exacerbating the scale of varices. Balloon-occluded retrograde transvenous obliteration (BRTO) is a widespread and well-reported treatment, whether preventive or emergency, for solitary varices derived from gastrorenal shunts (GRS) [5].

Situs inversus totalis (SIT) is a congenital abnormality that cause mirror image positioning of thoracic and abdominal organs [6]. In particular, in situs inversus abdominus (SIA) the abdominal organs below the diaphragm are laterally inverted. The incidence of SIA is approximately one in 100,000 people [6]. Since organs and vessels are not perfectly symmetrical in SIA, recognizing the anatomy of organs vessels is difficult, leading to diagnosis and treatment difficulties.

Owing to the low frequency of SIA, there have been no reports of BRTO for GVs in SIA cases. Recognizing the vascular anatomy before treatment contributes to success of the procedure. Herein, we report an SIA case of BRTO and partial splenic embolization (PSE) for GVs.

## Case report

**Fig. 1:**
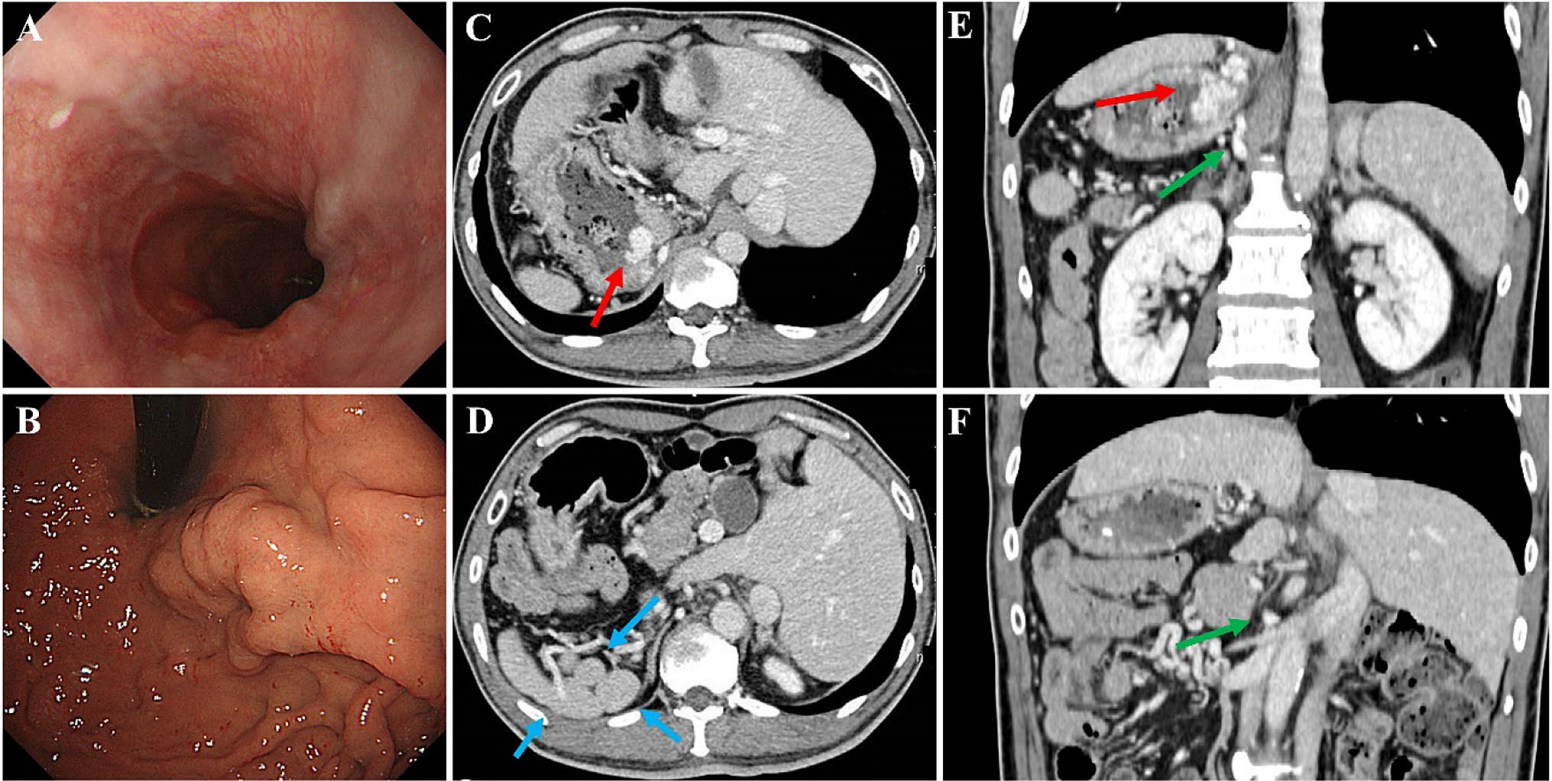
Esophagogastroduodenoscopy and computed tomography at the pre-balloon-occluded retrograde obliteration. **a** Esophagogastroduodenoscopy showing F1-shaped esophageal varices (Li, RC0). **b** Esophagogastroduodenoscopy showing F2-shaped gastric varices (GVs) (Lg-c, RC0). **c**, **d** Axial sections of abdominal computed tomography showing situs inversus abdominus, GVs (red arrow), and polysplenia (blue arrows). **e**, **f** Coronal sections of abdominal computed tomography showing GVs (red arrow) and gastrorenal shunt (green arrows)

**Fig. 2:**
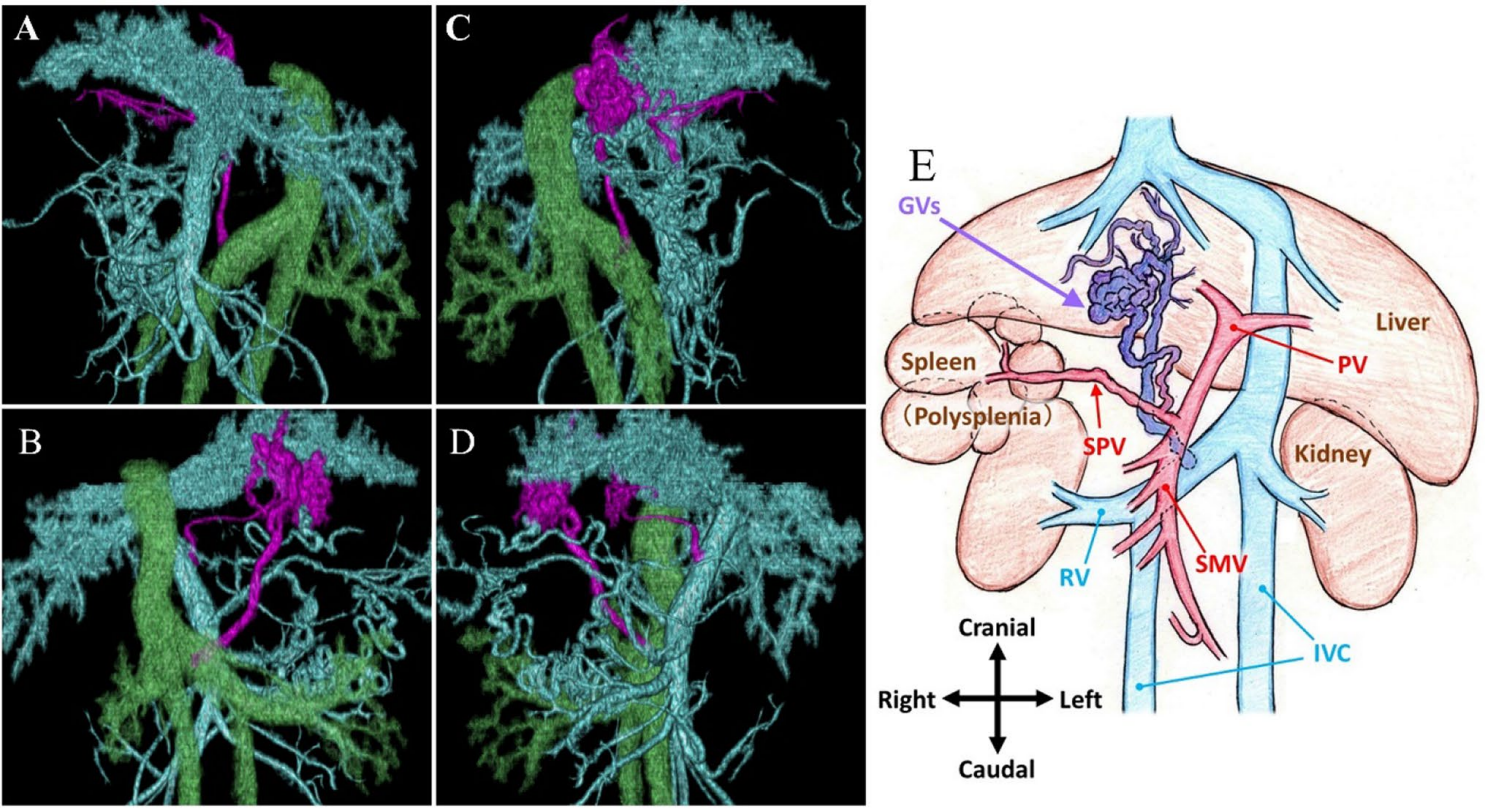
Computed tomography three-dimensional reconstruction images (3D CT). **a**–**d** 3D CT image showing the inferior vena cava (green), portal vein (blue), and gastrorenal shunt (purple) (**a** anterior to posterior; **b** posterior to anterior; **c** right to left; **d** left to right). **e** Schema of the main abdominal organs and vessels. *GVs* gastric varices, *IVC* inferior vena cava, *PV* portal vein, *RV* renal vein, *SMV* superior mesenteric vein, *SPV* splenic portal vein

A 55-year-old man with alcoholic cirrhosis presented with abdominal pain and underwent esophagogastroduodenoscopy showing F2-shaped GVs (Lg-cf, RC0) and F1-shaped EVs (Li, RC0) (Fig. 1a, b). The hepatic functional reserve was preserved (total bilirubin = 1.0 mg/dL, albumin = 4.1 g/dL, prothrombin time = 56%, no history of hepatic encephalopathy and ascites, Child–Pugh score 6, grade A). Abdominal dynamic computed tomography (CT) showed SIA, polysplenia, EVs, GRS, and GVs (Fig. 1c–f). Using CT three-dimensional reconstruction images (3D CT) (Fig. 2a–e), the vascular anatomy was carefully confirmed. The inferior vena cava (IVC) formed a right and left duplication type. The GRS merged into the right renal vein and the right renal vein merged into the right IVC. Since emergency interventional radiology during GV bleeding was extremely difficult due to the complex anatomy, prophylactic treatment (BRTO and PSE) with careful preparation was expected to be useful. Therefore, we gave the patient a through explanation of the condition and treatment, and we recommended the prophylactic treatment. The patient provided written informed consent.

**Fig. 3:**
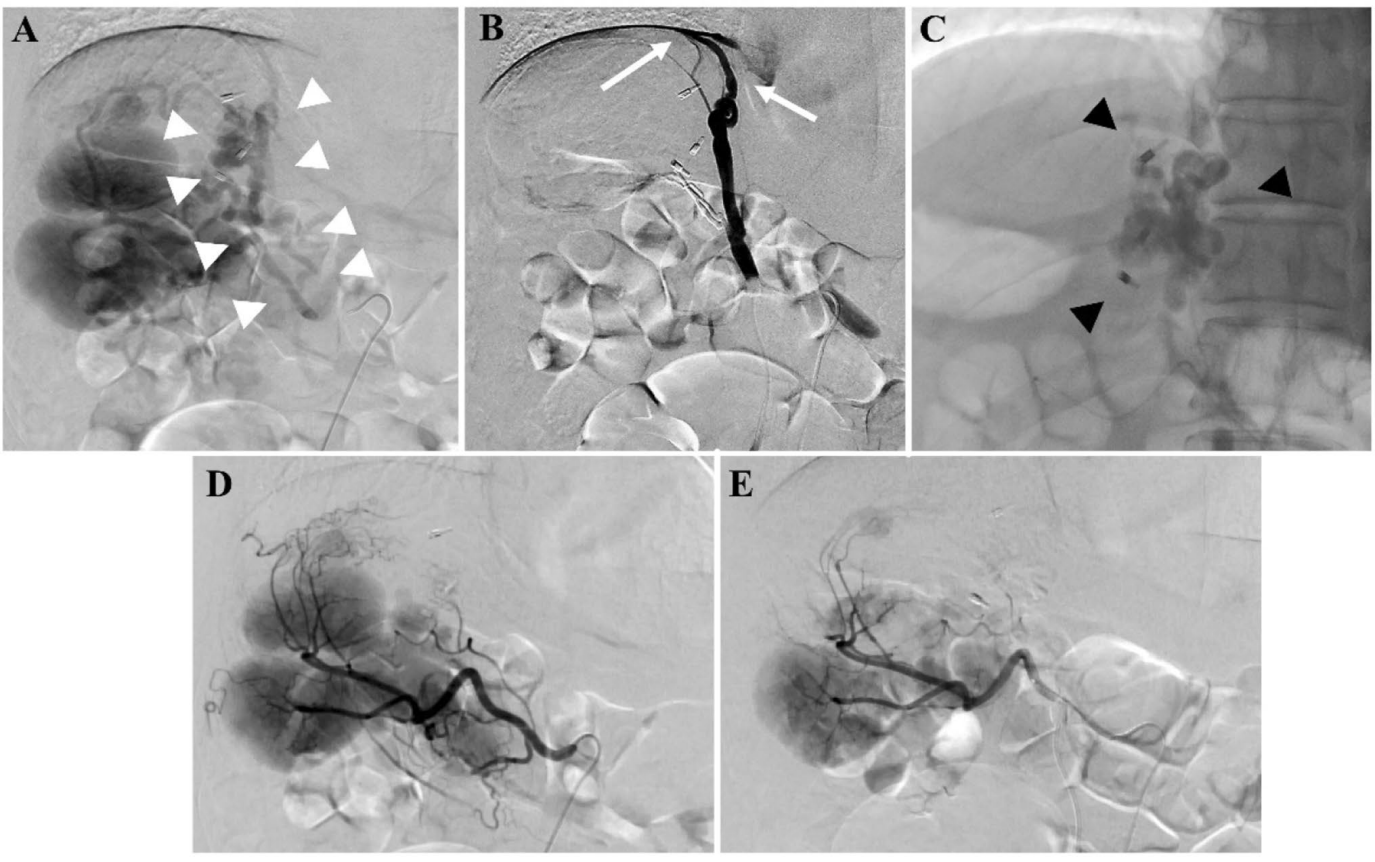
Digital subtraction angiography during balloon-occluded retrograde transvenous obliteration and partial splenic embolization (PSE). **a** The portal phase of splenic arteriography showing the gastric varices (GVs) and gastrorenal shunt (white arrowheads). **b** Balloon-occluded retrograde transvenous venography showing the right inferior phrenic vein (white arrows) as a draining vessel. The GVs are not visualized. **c** GVs are filled with 5% ethanolamine oleate with iopamidol. **d**, **e** The arterial phase of splenic angiography before PSE showing polysplenia (**d**) and after PSE showing polysplenia with partial embolism (**e**)

**Fig. 4:**
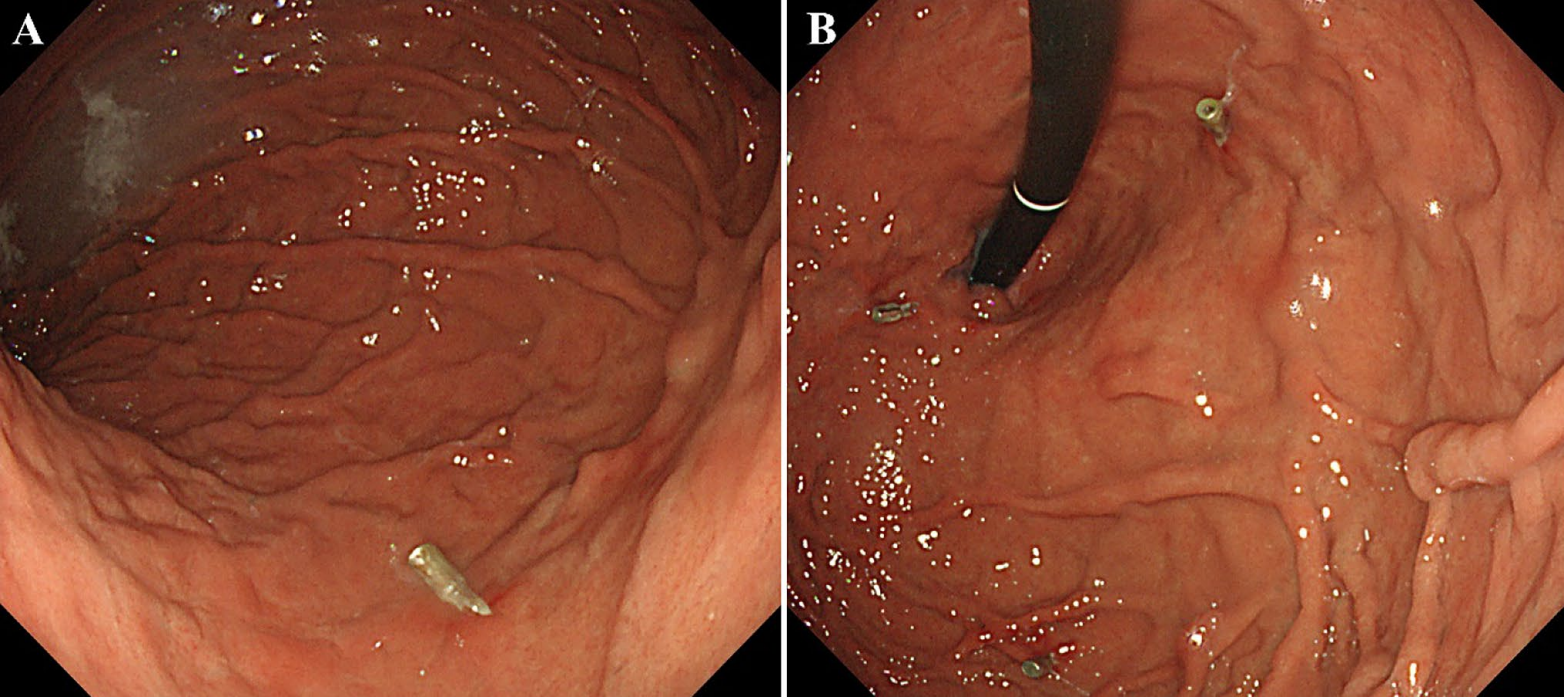
Esophagogastroduodenoscopy post balloon-occluded retrograde transvenous obliteration (BRTO). **a**, **b** The gastric varices have disappeared 3 months after BRTO

To evaluate the precise hemodynamic characteristics of GRS, splenic arteriography was performed first. A 5-Fr shepherd hook-type catheter (GoodTec, Goodman, Aichi, Japan) was introduced to the splenic artery via the left femoral artery. The portal phase of splenic arteriography showed a GRS, afferent veins (posterior gastric and left gastric veins), and an efferent vein (right renal vein) as shown in the 3D CT (Fig. 3a). An 8-Fr straight-type sheath (Radifocus introducer II H, Terumo, Tokyo, Japan) was introduced to the right IVC through the right femoral vein. A 5.2-Fr catheter with an occlusive balloon 9 mm in diameter (Selecon MP Catheter, Terumo Clinical Supply, Gifu, Japan) was advanced into the GRS. Balloon-occluded retrograde transvenous venography revealed the right inferior phrenic vein as a draining vessel, and the gastric varices were not visualized (Fig. 3b). Due to slow blood flow in the inferior phrenic vein and a contrast delay, 10 mL of 50% dextrose solution and 5% ethanolamine oleate and iopamidol (5% EOI) was administered stepwise to the inferior phrenic vein under balloon occlusion. In addition, the gastric varices were filled with an additional 10 mL of 50% dextrose solution and 7 mL of 5% EOI and remained stagnant in the vessels overnight (Fig. 3c). Following overnight occlusion, the catheter was removed after confirming that the blood flow in the GRS had disappeared. To prevent renal dysfunction secondary to hemolysis caused by EOI, haptoglobin (4000 units) was administered intravenously before EOI injection. Furthermore, to decrease the portal venous pressure after BRTO, PSE was performed for polysplenia (Fig. 3d, e). A 2.7-Fr microcatheter (Masters HF Catheter, Asahi Intecc, Aichi, Japan) was advanced into the hilum of the spleen along with a hook-type catheter. During PSE, the arterial vascular anatomy was just as complex as the venous one. Approximately 50% PSE involving gelatin sponge pieces cut into 3 mm cubes was performed, paying close attention to prevent embolism to other organs. To prevent splenic abscess, antibiotics (imipenem hydrate/Cilastatin sodium, 1.0 g daily) were administered intravenously before PSE and for a week after treatment. The patient was discharged 15 days after BRTO without any adverse events such as splenic abscess, portal vein thrombosis, or hemoglobinuria. The GVs were confirmed to have disappeared 3 months after BRTO (Fig. 4a, b). There was no portal vein thrombosis, decreased hepatic functional reserve, exacerbation of EVs, or ectopic varices.

## Discussion

The efficacy of BRTO in GVs with GRS is well established and widely accepted as a treatment [5]. A retrospective study of BRTO showed that the clinical success rate of BRTO—that is, no recurrence of GVs with no rebleeding—was 97.3%, confirming the efficacy of BRTO [7]. The combination of BRTO with PSE can decrease the portal venous pressure and prevent the exacerbation of EVs [8–12], and can significantly reduce the amount of the sclerosing agent required, including 5% EOI for BRTO [8]. However, exacerbation of EVs has been observed in 33.3% of patients treated with BRTO without PSE [7]. Based on the results of these clinical studies, we simultaneously performed BRTO with PSE.

Although there is right-to-left reversal of organs in SIA, organ function is almost normal. However, SIA is also associated with various organ malformations; the most frequent being cardiovascular malformations, followed by the lack of an ascending colon, biliary malformations, diaphragmatic laxity, teeth defects, and polysplenia [13]. In this case, the patient had polysplenia and a complex vascular anatomy associated with vascular malformations. As mentioned, there are no reports of BRTO for GVs in SIA; however, there are some reports of surgical procedures (especially cholecystectomy) for SIT or SIA patients [14–16]. In those reports, the following three points were important for the success of the procedure: (i) sufficient preoperative discussion about the anatomy; (ii) reversal positioning of the surgeon, assistants, instruments, and monitors; and (iii) operation with care to avoid misidentification of the anatomy. It is important to visualize the organ position preoperatively and to perform a sufficient simulation of the procedure [17, 18]. These factors also apply to the BRTO procedure. Sufficient visualization of the anatomy, and repeated simulation of the procedure before BRTO contribute to success.

The most important anatomical factors for BRTO in this case were the afferent and efferent veins of GVs. An accurate assessment of the vascular anatomy and diameter of the efferent vein, using a straight-type sheath and a catheter with an occlusive balloon 9 mm in diameter, was the best way to perform BRTO. It was difficult to insert a microcatheter selectively to the targeted efferent vessels of GVs because they were so small and could not be visualized by preoperative CT. Therefore, the stepwise sclerosant injection method was used for embolizing the small efferent vessels of GVs. Despite this technical difficulty, the BRTO was effective for GVs.

Since SIA is a congenital abnormality, the anatomy of the organs and blood vessels vary individually depending on the stage or location of the embryonic period. In this case, the IVC formed a right and left duplication type as a vascular malformation and was not completely symmetrical. Thus, since there is no fixed anatomical type, accurate understanding of the anatomy of organs and blood vessels before treatment is essential for success.

Since there have been no reports of BRTO for GVs in SIA cases, the detailed organ and vascular anatomy of SIA obtained from this case is important. This case will serve as a reference for successful treatment in future cases.

## Abbreviations

BRTO: Balloon-occluded retrograde transvenous obliteration
PSE: Partial splenic embolization
CT: Computed tomography
GVs: Gastric varices
EVs: Esophageal varices
GRS: Gastrorenal shunt
SIT: Situs inversus totalis
SIA: Situs inversus abdominus
3D CT: Computed tomography three-dimensional reconstruction images
